# Caffeine and Doping—What Have We Learned since 2004

**DOI:** 10.3390/nu12082167

**Published:** 2020-07-22

**Authors:** Patrick Diel

**Affiliations:** Department Molecular and Cellular Sports Medicine; Center of Preventive Dopoing Research, German Sports University Cologne, 50933 Cologne, Germany; diel@dshs-koeln.de

Caffeine is a naturally occurring plant alkaloid and is found in plant constituents such as coffee and cocoa beans, tea leaves, guarana berries and the kola nut [1]. It is added to a variety of foods, such as baked pastries, ice creams, sweets, and cola drinks. In energy drinks, caffeine is frequently combined with substances such as taurine and D-glucurono-γ-lactone [2]. Moreover, it belongs to the most frequently used and consumed pharmacologic substances [3]. Caffeine exerts a variety of effects on human physiology. Caffeine is absorbed rapidly in the gastrointestinal tract about 40 to 60 minutes after uptake peak plasma concentrations are reached [4], and, after 3 to 5 hours, 50% has been excreted [5]. As a lipophilic substance it is able to cross membranes such as the blood–brain barrier or placenta [6]. Caffeine uptake results in ergogenic effects. In humans, caffeine stimulates the central nervous system, and in moderate doses increases alertness and reduces sleepiness [1]. In sports, it has been shown that caffeine is ergogenic and improves athletic performance, especially endurance performance involved in sports such as running, swimming and cycling [7,8]. Here, its consumption has been shown to increase in the duration of the exercise or a decreased perception of exertion [1,9]. Moreover, the effects on anaerobic-based performance and strength performance have been demonstrated [10]. These performing enhancing effects are also confirmed by the International Olympic Committee in its consensus statement on dietary supplements [11]. Based on this information, it is not surprising that caffeine is present in a huge number of food supplements that are marketed for weight loss and sports performance [2].

Between 1984 and 2004, caffeine was a banned substance in sports and its use was prohibited in competition. Despite the fact that the scientific evidence for performance enhancing effects of caffeine is increasing over the years, the World Anti-Doping Agency (WADA) decided to remove caffeine from the list of banned substances with effect from January 1, 2004, and moved it to the so called monitoring program. 

The consumption of caffeine-containing products in the general population has significantly increased in recent years, especially in the field of public sports [12]. This is due to the rise of new sports disciplines, such as, for example, E-sports and the advertising and promotion of caffeine-containing products in relation to public sports. Simultaneously, there is a rise in concern about the safety of caffeine consumption in the general population and in specific groups, such as adults performing physical activity, and individuals consuming caffeine together with alcohol or substances found in energy drinks [2].

Against this background and based on the fact that, on the one hand, there is increasing scientific evidence that caffeine uptake results in performance-enhancing effects and, on the other hand, athletes today are able to consume caffeine-containing products freely, it is of high interest to elucidate whether the use of such products has increased in performance sports since 2004. A recently published paper provides data suitable to answer this relevant question [13]. In this study, the authors analyze the caffeine concentrations in more than 7000 urine samples obtained in official competitions held in Spain from the years 2004, 2008 and 2015. Males and females and different sports disciplines were included in the analysis. The authors detected a significant increase in the percentage of samples with detectable caffeine between 2004–2008 and 2015. Their investigations also reveal that there are significant differences between different sports disciplines in the consumption of caffeine. The highest urine caffeine concentrations were detected in disciplines having an aerobic-like nature, such as cycling, athletics and rowing. The highest increases between 2004–2008 and 2015 were detected in aquatics, but also disciplines relevant for public sports, such as football [13].

Reviewing these data, it is obvious that the removal of caffeine from the list of banned substances has not resulted is a strong increase in the consumption of caffeine, at least in the analyzed population of athletes and in Spain. However, the performance-enhancing effect of caffeine is believed to be dependent on a variety of factors, such as individual genetics [14,15], microbiome [16], nutrition [17], combination with other substances [12] and also the nature of performance [13]. Therefore, it is likely that the uptake of caffeine may not result in all athletes in an enhancement of performance, and this could explain the moderate increase in the total of all analyzed samples. Nevertheless, the data from Navaro et. all show a clear trend in an increased use of caffeine in athletes in the monitoring program. Taking in account that the use of caffeine in athletes that are not participating in the monitoring program and in public sports may also follow this trend, we have to conclude that a significant and increasing number of people worldwide consume caffeine with the aim to enhance performance. In these individuals, a high uptake of the substance meets high-intensity exercise. This is alarming in relation to the concerns about caffeine related to safety and possible adverse effects. The European food safety administration (EFSA) has published in 2015 a Scientific Opinion on the safety of caffeine [2]. EFSA has come to the conclusion that habitual caffeine consumption of up to 400 mg per day does not give rise to safety concerns for non-pregnant adults. Habitual caffeine consumption of up to 200 mg per day by pregnant women does not give rise to safety concerns for the fetus. Single doses of caffeine and habitual caffeine intakes of up to 200 mg consumed by lactating women do not give rise to safety concerns for breastfed infants. This seems to be good news; however, in relation to sports, it is of relevance that an increasing consumption of caffeine, especially in public sports, is via energy drinks. Energy drinks consumed for the uptake of caffeine are no sports drinks. They are hypertonic because they contain high concentrations of sugar [12], resulting in a slower uptake of water. Moreover, the high sugar concentrations may result in a higher thirstiness resolution in a higher consumption of such drinks and a least in a high uptake of caffeine [12]. There are no indications that an uptake of up to 200 mg also in combination with intensive physical performance does increase individual health risk (2). However, the risk of higher concentrations of caffeine in combination with physical activity is uncertain and there is a variety of safety concerns in relation to several physiological and pathologic scenarios [18]. Moreover, it is very likely that individuals consuming caffeine with the aim to enhance performance consume much higher amounts than 200 mg a day.

In conclusion, the data provided by Navarro et al., 2019, provide indications that the use of caffeine in elite sports has increased after its removal from the list of banned substances 2004. There is also increasing evidence that the ergogenic activity of caffeine and individual genetics, metabolics and variables determine these effects. Moreover, the safety of caffeine, at least at high doses and in combination with other substances, is still under debate. Therefore, further research is needed in this field. In the future, the monitoring of caffeine consumption in elite sports during doping control needs to be continued. 
